# CD248, targeted by veratramine and neobavaisoflavone, mediates pathological changes of renal tubular epithelial cells induced by high glucose

**DOI:** 10.1186/s41065-025-00624-z

**Published:** 2025-12-12

**Authors:** Mei Lin, Nan Hu, Zhen Wang, Ping Li, Dan Song, Xinzhou Zhang

**Affiliations:** 1 Department of Nephrology, Shenzhen People’s Hospital (The Second Clinical Medical College, Jinan University; The First Affiliated Hospital, Southern University of Science and Technology), Shenzhen, Guangdong 518020 China; 2 Department of Nephrology, Shenzhen Guang Ming District People’s Hospital, ShenZhen, Guangdong 518000 China

**Keywords:** Diabetic nephropathy, CD248, TGF-β1/Smad pathway, Veratramine, Neobavaisoflavone

## Abstract

**Background:**

Epithelial-mesenchymal transition (EMT) of tubular epithelial cells are one of the major pathological changes of diabetic nephropathy (DN). Cluster of differentiation 248 (CD248) has been reported to be associated with fibrosis after kidney injury. The aim of this study was to investigate the mechanism of CD248 in DN and its targeted compounds.

**Materials and methods:**

Virtual screening, molecular docking and Cellular thermal shift assays were used to explore potential small molecule compounds targeting CD248. In vitro DN model was established by treating human proximal renal tubular epithelial cell line HK-2 with high glucose (HG), and db/db mice were used as the animal model. siRNA transfection was used to knockdown CD248 in HK-2 cells, and HK-2 cells and the animals were treated with veratramine (VER) or neobavaisoflavone (NBIF). qPCR was used to detect the mRNA expression of CD248, tumor necrosis factor-α (TNF-α), interleukin (IL)-6 (IL-6), and IL-1β. Western blot was used to assess protein expression level of CD248, EMT-associated proteins, fibrosis markers, and TGF-β1/Smads pathway-associated proteins. CCK-8 assay and flow cytometry were used to detect cell viability and apoptosis, respectively. Histopathological and various biochemical indicators were used to assess renal injury in animals.

**Results:**

CD248 was significantly up-regulated in HG-induced HK-2 cells. CD248 knockdown inhibited HG-induced cell proliferation inhibition, apoptosis and inflammatory response. HG stimulation significantly reduced the protein expression level of E-cadherin in HK-2 cells, and increased the expression levels of vimentin, α-smooth muscle actin (α-SMA), collagen I, collagen IV, fibronectin, TGF-β1, p-Smad2, p-Smad3, and Smad4, while CD248 knockdown reversed these effects. In addition, VER and neobavaisoflavone were found to bind with CD248, and they inhibited HG-induced apoptosis, inflammation, EMT and extracellular matrix synthesis in HK-2 cells, and ameliorate the renal injury of db/db mice. VER and NBIF also inhibited HG-induced activation of TGF-β1/Smads axis.

**Conclusion:**

CD248 participates in HG-induced EMT of renal tubular epithelial cells and renal fibrosis by regulating TGF-β1/Smads pathway, and VER and NBIF are two potential natural drugs which targets it to ameliorate DN.

**Supplementary Information:**

The online version contains supplementary material available at 10.1186/s41065-025-00624-z.

## Introduction

 Diabetes mellitus (DM) has become one of the biggest health problems of the 21 st century, affecting an estimated 463 million adults worldwide, and the global prevalence of DM is expected to reach 700 million by 2045 [1–3]. DM leads to a series of complications, including diabetic retinopathy, diabetic neuropathy and diabetic nephropathy (DN) [4]. About one-third of DM patients will develop DN, which is one of the main causes of end-stage renal disease (ESRD) [5]. At present, the treatment of DN is mainly to control glucose level, reduce blood pressure, reduce urinary protein, control hyperlipidemia, etc [6]. However, few drugs have been approved in the past several decades specifically to prevent DN or improve kidney function, due to poor understanding of the mechanisms of progressive renal dysfunction [7]. There is an urgent need to improve understanding of the pathogenesis of DN to facilitate the development of novel, effective and safe treatments.

Renal tubulointerstitial fibrosis is a key pathological change in the progression of DN, and is characterized by the deposition of extracellular matrix (ECM), including fibronectin, collagen I and so on [8, 9]. Epithelial-mesenchymal transformation (EMT) of renal tubular epithelial cells may be associated with the progression of DN, accompanied with the loss of epithelial phenotype and the acquisition of pro-fibrotic features [10, 11]. Transforming growth factor β1 (TGF-β1) is a key mediator in the development of renal fibrosis, and EMT and ECM accumulation in DN are induced and maintained by TGF-β1/Smad pathway [10, 12, 13]. Therefore, interventions targeting TGF-β1/Smad signaling pathways may become a new strategy for DN treatment.

Cluster of differentiation 248 (CD248), also known as endosialin or tumor endothelial marker 1(TEM-1), is a type 1 transmembrane glycoprotein [14]. CD248 plays a role in embryonic development and is not expressed or lowly expressed in adult tissues [15]. However, CD248 mRNA expression increases and persists in the kidney, especially in mesangial cells and perivascular cells [16]. Recently, single-cell RNA sequencing studies have shown that CD248 is specifically enriched in renal tubular epithelial cells and stromal cells of DN patients [17]. CD248 has been reported to be a therapeutic target for cancers and fibrotic diseases. CD248 is highly expressed in ossteosarcoma tissues, and its high expression is associated with lung metastasis; knockdown of CD248 can inhibit the migration and invasion of osteosarcoma cells [18]. CD248 is up-regulated in myofibroblasts of mouse models of renal and peritoneal fibrosis, and CD248 knockdown can alleviate renal and peritoneal fibrosis [19]. CD248 also promotes insulin resistance by binding to insulin receptors and inhibiting their insulin-induced autophosphorylation [20]. In addition, CD248 is upregulated in the glomeruli of diabetic mice, and CD248 deficient mice lack pathological phenotypes, which implies that targeting CD248 improves glomerular dysfunction and blocks DN progression [21]. However, the function and mechanism of CD248 in renal fibrosis and EMT of DN remain largely unknown.

Here, we aim to explore the effects of CD248 on high glucose (HG) -induced EMT and pro-fibrotic phenotypes of human proximal renal tubular epithelial cell line HK-2. In this study, it was observed that CD248 knockdown inhibited EMT and pro-fibrotic phenotypes of HG-induced HK-2 cells and inhibited TGF-β1/Smad signaling pathway. More importantly, veratramine (VER) and neobavaisoflavone (NBIF) were identified as natural compounds targeting CD248, which inhibited HG-induced EMT and pro-fibrotic phenotypes of HK-2 cells, and ameliorated renal injury of db/db mice. This study brings new insights into the prevention and treatment of DN.

## Materials and methods

### Cell culture and treatment

HK-2 cells were purchased from the American Typical Culture Preservation Center (ATCC; Manassas, VA, USA). In an incubator (37℃ and 5% CO_2_), HK-2 cells were cultured in Dulbecco’s Modified Eagle’s Medium (DMEM; Thermo Fisher Scientific, Carlsbad, MA, USA) supplemented with 10% fetal bovine serum (FBS; Thermo Fisher Scientific, Carlsbad, MA, USA), 100 U/mL penicillin and 100 µg/mL streptomycin (Beyotime, Shanghai, China). When HK-2 cells grew to about 80% confluency, the cells were cultured in serum-free medium overnight, and then cultured with normal glucose (NG, 5.5 mM glucose), mannitol (5.5 mM glucose + 19.5 mM mannitol) or HG (25 mM glucose) for 48 h [22].

For TGF-β1 activator treatment, HK-2 cells were pretreated with SRI-011381 hydrochloride (10 µM; MedChemExpress, Shanghai, China) [23] for 2 h, then transfected with si-CD248#1 or si-NC, and finally treated with HG for 48 h.

VER (purity > 98%) and NBIF (purity: 99.91%) were purchased from MedChemExpress (Shanghai, China). To evaluate the cytotoxic effects of VER and NBIF on HK-2 cells, HK-2 cells were treated with different concentrations of VER (0, 1.25, 2.5, 5, 10, and 20 µM) or different concentrations of NBIF (0, 5, 10, 20, 40, and 80 µM) for 24 h under NG condition. To investigate the effects of VER or NBIF on HG-induced EMT and pro-fibrotic phenotype of HK-2 cells, HK-2 cells were pretreated with 10 µM and 20 µM VER or 10 µM and 40 µM NBIF for 24 h and then treated with 25 mM glucose for 48 h.

### Cell transfection

Small interfering RNA (siRNA) negative control (si-NC) and siRNAs against CD248 (si-CD248#1, si-CD248#2) were purchased from the GenePharma Co.,Ltd. (Shanghai, China). Transient transfection was performed on a 6-well plate using Lipofectamine™ 2000 reagent (Thermo Fisher Scientific Inc., Waltham, MA, USA) according to the manufacturer’s instructions. 48 h after transfection, cells were collected for validation of the knockdown efficiency and subsequent experiments.

### Reverse transcriptional quantitative polymerase chain reaction (qPCR)

Total RNA of HK-2 cells and kidney tissues was extracted using a TRIzol reagent kit (Invitrogen, Carlsbad, CA, USA). The extracted RNA was reverse-transcribed into cDNA using the PrimeScript™ RT reagent kit (Takara, Dalian, China). qPCR was performed using a 2×SYBR Green RT-qPCR Master Mix kit (Selleckchem, Houston, TX, USA) in the CFX Connect real-time PCR detection System (Bio-Rad, Hercules, CA, USA). The PCR cycle conditions used were: 95℃ for 2 min, 40 cycles of 95℃ for 30 s, 60℃ for 30 s and 72℃ for 60 s. GAPDH was used as an internal reference gene, and the relative expression levels of the target genes were calculated by 2^−△△CT^ method. The primers were synthesized by GenePharma (Shanghai, China). Primer sequences are shown in Table 1.

**Table 1:** Sequences of the primers used for qPCR

Gene	Human	Mouse
CD248	Forward: 5’-GTCCCCTACCACTCCTCAGT-3’	Forward: 5’-GCTACCTCTGCCAGTTTGGT-3’
	Reverse: 5’-CATGGGTTCTGTTGGGCTCT-3’	Reverse: 5’-GAAGGCTGTTTCACGCACAG-3’
TNF-α	Forward: 5’-GTGACAAGCCTGTAGCCCAT-3’	Forward: 5’-ACCCTCACACTCACAAACCA-3’
	Reverse: 5’-CAGACTCGGCAAAGTCGAGA-3’	Reverse: 5’-ATAGCAAATCGGCTGACGGT-3’
IL−6	Forward: 5’-TCCACAAGCGCCTTCGGTC-3’	Forward: 5’-CCCCAATTTCCAATGCTCTCC-3’
	Reverse: 5’-GGTCAGGGGTGGTTATTGCAT-3’	Reverse: 5’-CGCACTAGGTGTGCCGAGTA-3’
IL-1β	Forward: 5’-AACCTCTTCGAGGCACAAGG-3’	Forward: 5’-GACAGGTCAGTGGGTACTGG-3’
	Reverse: 5’-AGATTCGTAGCTGGATGCCG-3’	Reverse: 5’-TAAGTGGGTGCTCTGGTTGC-3’
GAPDH	Forward: 5’-CACTAGGCGCTCACTGTTCT-3’	Forward: 5’-CTTCTCCTGCAGCCTCGT-3’
	Reverse: 5’-TTCCCGTTCTCAGCCTTGAC-3’	Reverse: 5’-ACTGTGCCGTTGAATTTGCC-3’

### Western blot

HK-2 cells and kidney tissues were lysed in RIPA lysis buffer (Beyotime, Shanghai, China) and the protein concentration was detected with a bicinchoninic acid (BCA) protein detection kit (Solarbio, Beijing, China). The protein (20 µg) in each sample was isolated via sodium dodecyl sulphate-polyacrylamide gel electrophoresis (SDS-PAGE) and transferred to the polyvinylidene fluoride (PVDF) membranes (Millipore, Billerica, MA, USA). After blocking the film with 5% skim milk at room temperature for 1 h, the membrane was incubated with the primary antibodies including anti-CD248 antibody (Cell signal technology, #38774, 1:1000), anti-E-cadherin antibody (Abcam, ab227639, 1:1000), anti-Vimentin antibody (Abcam, ab16700, 1:1000), anti-alpha-smooth muscle actin (α-SMA) antibody (Abcam, ab314895, 1:1000), anti-collagen I antibody (Abcam, ab316222, 1:1000), anti-collagen IV antibody (Abcam, ab214417, 1:1000), anti-fibronectin antibody (Abcam, ab2413, 1:1000), anti-TGF-β1 antibody (Abcam, ab215715, 1:1000), anti-Smad2 antibody (Abcam, ab280888, 1:1000) 1:1000), anti-phospho (p)-Smad2 antibody (Abcam, ab280888, 1:1000), anti-Smad3 antibody (Abcam, ab84177, 1:1000), anti-p-Smad3 antibody (Abcam, ab52903, 1:1000), anti-Smad4 antibody (Abcam, ab40759, 1:1000), anti-Smad1/5/9 antibody (Abcam, ab300164, 1:1000), anti-p-Smad15/9 antibody (Abcam, ab92698, 1:1000), anti-Smad7 antibody (Abcam, ab216428, 1:1000), and anti-GAPDH antibody(Abcam, ab181602, 1:1000), overnight at 4 °C. After washing the membranes with tris buffered saline tween (TBST), the membranes were then incubated at 37 °C for 1 h with the horseradish peroxidase (HRP) -coupled secondary antibody (Abcam, ab6721, 1:500). The membranes were then washed with TBST again, and the A solution and B solution of an electrochemical luminescence (ECL) kit (Pierce Biotechnology, Rockford, IL, USA) were mixed, and added onto the membranes. The the chemiluminescence signal was monitored and quantified by the iBright software (Invitrogen, Carlsbad, CA, USA).

### Cell proliferation assay

The proliferation of HK-2 cells was assessed with a cell counting Kit-8 (CCK-8) kit (Beyotime, Shanghai, China). Briefly, HK-2 cells at logarithmic growth stage were inoculated into 96-well plates with 5 × 10^3^ cells per well and cultured overnight at 37℃. 10 µl CCK-8 solution was added to each well at the specified time point (0, 24, 48 and 72 h) and incubated at 37℃ for 2 h. Next, the absorbance (optical density, OD) value of each well was detected at 450 nm using a microplate reader (ThermoFisher Multiskan FC, Waltham, MA, USA). Cell viability was calculated using the following formula: cell viability = (OD of treated cells-OD of blank)/(OD of control-OD of blank) × 100%.

### Apoptosis detection

An Annexin V-Fluorescein Isothiocyanate (FITC)/propidium iodide (PI) apoptosis detection kit (Beyotime, Shanghai, China) was used to detect the apoptosis of HK-2 cells. HK-2 cells were collected and washed with cold sterile phosphate buffer saline (PBS) twice. The cells were then re-suspended at room temperature in a 100 µL binding buffer containing 5 µL Annexin V-FITC and 5 µL PI, and stained in the dark for 30 min. After PBS washing, the stained cells were analyzed using a FACScan flow cytometer (BD Biosciences, San Jose, CA, USA) within 1 h, and the rate of apoptosis was analyzed using FlowJo V.10 software.

### Virtual screening and molecular docking

The Natural Product Library (https://www.tsbiochem.com/library/natural_product_library) was applied for virtual screening. The Prep Wiz module of Schrödinger software package was used to process CD248 protein with hydrogenation and water removal. All compounds were initially screened for drug-likeness properties based on Lipinski’s rule of five with Veber rule modules in DS4.0, and molecules with poor drug-likeness properties were eliminated. The remaining molecules were prepared with the LigPrep module, the force field used the OPLS_2005 force field, and the protonation of the molecules was performed with the Epik module under pH7.4 conditions. The SiteMap module of Schrödinger software was used to predict the active pocket of CD248 protein. Then, the optimal docking condition Glide force field was selected and the protein file was used as the receptor for docking analysis. The active pocket of the protein receptor is defined as A size 10 Å × 10 Å × 10 Å pocket. Glide algorithm was used for docking with standard docking accuracy, and other parameters remain default.

### Cellular thermal shift assay (CETSA)

HK-2 cells were trypsinized, resuspended and inoculate, then treated with 20 µM VER, 40 µM NBIF, or an equal volume of dimethyl sulfoxide (DMSO; Beyotime, Shanghai, China) at 37 °C for 4 h, respectively. After treatment, the cells were washed with PBS and collected. The cell suspension was aliquoted into multiple PCR tubes and subjected to heat shock at different temperatures (ranging from 37 °C to 62 °C, in 5 °C increments) for 3 min using a quantitative PCR instrument. Subsequently, the cells were rapidly cooled on ice and underwent three freeze-thaw cycles (alternating between liquid nitrogen and a 37 °C water bath) to achieve complete cell lysis. The lysates were centrifuged at 4 °C and 20,000 × g for 20 min to isolate the soluble protein fraction. Finally, the content of CD248 protein in the supernatant was detected by Western blot analysis.

### Animal model

Male db/db mice (C57BLKS/J-leprdb/leprdb, *n* = 24) at 6 weeks of age and matched littermate db/m mice (C57BLKS/J-leprdb/leprm, *n* = 6, serving as normal controls) were housed under a 12 h light/dark cycle at a temperature range of 20℃ to 24℃ with 60% relative humidity and allowed free access to water and food. The db/db mice were randomly assigned to the following groups (*n* = 6 per group): DN, DN + VER, and DN + NBIF. Mice in the DN + VER and DN + NBIF groups received 20 mg/kg VER or 40 mg/kg NBIF, respectively, via oral gavage daily. The animal in the DN group and normal control group were administered an equal volume of normal saline via oral gavage. All treatments were administered once daily for 12 consecutive weeks. All animal experiments were conducted in accordance with the Guide for the Care and Use of Laboratory Animals and were approved by the Animal Ethics Committee of Shenzhen People’s Hospital.

### Sample collection and biochemical analysis

Starting from week 3 and every two weeks thereafter, whole blood was collected from the tail vein of mice after an overnight fast, and fasting blood glucose (FBG) levels were measured using a portable glucose meter. Body weight was measured every two weeks starting from the third week. Additionally, after 12 weeks of treatment, 24-h urine samples were collected from each mouse using metabolic cages, rapidly frozen in liquid nitrogen, and stored at −80 °C. Urinary albumin levels were quantified using a commercial ELISA kit (ab108792, Abcam, Shanghai, China). At the end of the experiment, mice were anesthetized by intraperitoneal injection of 2,2,2-tribromoethanol, and whole blood samples were obtained via cardiac puncture. The samples were incubated in coagulant-promoting tubes at room temperature for 1 h and then centrifuged at 3000 × g for 15 min to collect the supernatant serum, which was stored at −80 °C. Serum creatinine and blood urea nitrogen (BUN) levels were assessed using commercial assay kits (Creatinine, C011-2-1; BUN, C013-2-1; Nanjing Jiancheng Bioengineering Institute, Nanjing, China), respectively. Kidney samples were rapidly excised and weighed. One kidney was flash-frozen in liquid nitrogen, while the other was fixed overnight in 4% paraformaldehyde and subsequently embedded in paraffin. All samples were stored at −80 °C. The kidney index was calculated as kidney weight (mg)/body weight (g).

### Histological analysis

Paraffin-embedded renal tissues were sectioned at a thickness of 4 μm and subjected to hematoxylin and eosin (H&E) staining. Renal structures were observed under a microscope (Zeiss AX10 microscope, Carl Zeiss Canada Ltd, Canada). Glomerular mesangial expansion and tubulointerstitial injury were evaluated and graded by randomly selecting 10 fields of view. The renal histopathological analysis was performed by two independent investigators in a blinded manner.

### Statistical analysis

All experiments were conducted independently in triplicate (*n* = 3 independent biological replicates, each performed with cells from different passages). SPSS 21.0 software (IBM Corp., Armonk, NY, USA) was used for statistical analysis. All data are expressed as mean ± standard deviation (SD). Comparisons between groups were made using Student’s t-test or one-way analysis of variance (ANOVA) and Tukey post-hoc test. *P* < 0.05 was considered statistically significant.

## Results

### Effect of CD248 knockdown on HG-induced apoptosis and inflammatory response of HK-2 cells

**Fig. 1:**
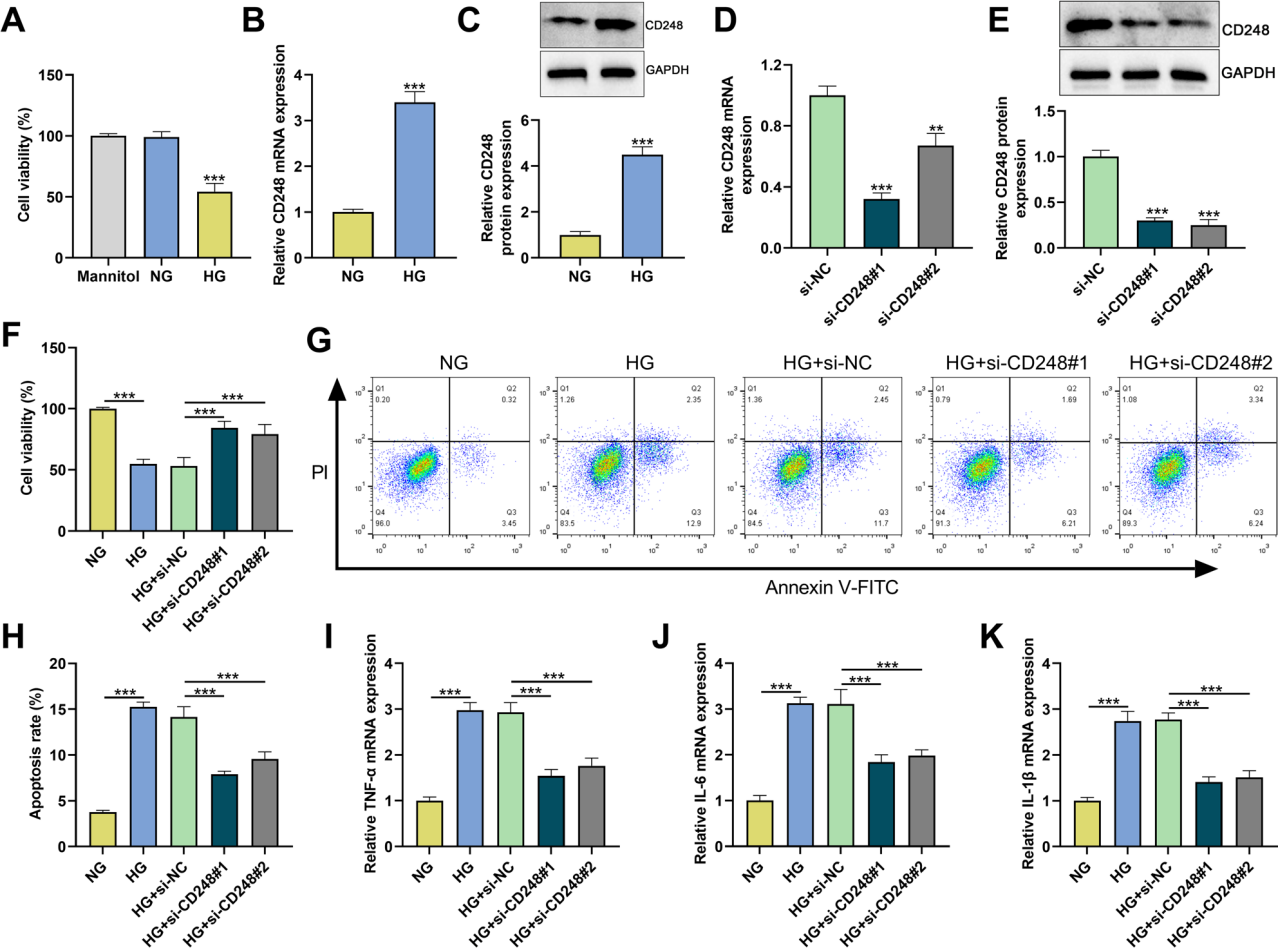
CD248 knockdown inhibited HG-induced apoptosis and inflammation of HK-2 cells. **A** After treating HK-2 cells with 5.5 mM glucose, 5.5 mM glucose + 19.5 mM mannitol, and 25 mM glucose for 48 h, the cell viability was detected using CCK-8. **B**&**C** After HK-2 cells were treated with high glucose (HG, 25 mM glucose) for 48 h, the mRNA and protein expression levels of CD248 were detected by qPCR (**A**) and Western blot (**B**), respectively. **D**&**E** mRNA and protein expression levels of CD248 in HK-2 cells transfected with si-NC, si-CD248#1 and si-CD248#2 were detected by qPCR (**C**) and Western blot (**D**), respectively. **F**-**K** HK-2 cells were treated with normal glucose (NG) or HG for 48 h after transfection with si-NC or si-CD248#1/2, and cell proliferation was detected by CCK-8 assay (**F**), and flow cytometry was used to evaluate apoptosis (**G**&**H**), and the mRNA expression levels of pro-inflammatory cytokines (TNF-α, IL-6 and IL-1β) were detected by qPCR (**I**-**K**). Data were presented as mean ± SD (*n* = 3 independent experiments). **P < 0.01, ***P < 0.001

To determine whether the DN cell model was successfully established, we first evaluated the effect of HG treatment on the viability of HK-2 cells. As shown in Fig. 1A, HG treatment significantly reduced the viability of HK-2 cells compared with the mannitol and NG groups. We then examined the expression level of CD248 in HK-2 cells stimulated by HG. qPCR and Western blot showed that the mRNA and protein expression levels of CD248 in HG-treated HK-2 cells were significantly up-regulated compared with NG group (Figs. 1B&C). In addition, transfection with si-CD248#1, si-CD248#2, or si-CD248#3 significantly reduced the mRNA and protein expression levels of CD248 in HK-2 cells compared to si-NC (Figs. 1D&E). Since si-CD248#1 and si-CD248#3 had higher knockdown efficiency, they were selected for subsequent assays. CCK-8 assay showed that HG treatment inhibited HK-2 cell viability, while CD248 knockdown partly reversed HG-induced inhibition of cell viability (Fig. 1F). Flow cytometry analysis showed that compared with NG group, the rate of apoptosis was significantly increased in HG group, and CD248 knockdown inhibited HG-induced apoptosis of HK-2 cells (Figs. 1G&H). In addition, compared with NG group, HG treatment significantly increased the mRNA expression levels of tumor necrosis factor-α (TNF-α), interleukin-6 (IL-6), and interleukin-1β (IL-1β) in HK-2 cells, while CD248 knockdown reversed this effect (Figs. 1I-K). These results suggest that CD248 knockdown inhibits HG-induced apoptosis and inflammation in HK-2 cells.

### Effect of CD248 knockdown on HG-induced EMT and pro-fibrotic phenotype of HK-2 cells

**Fig. 2:**
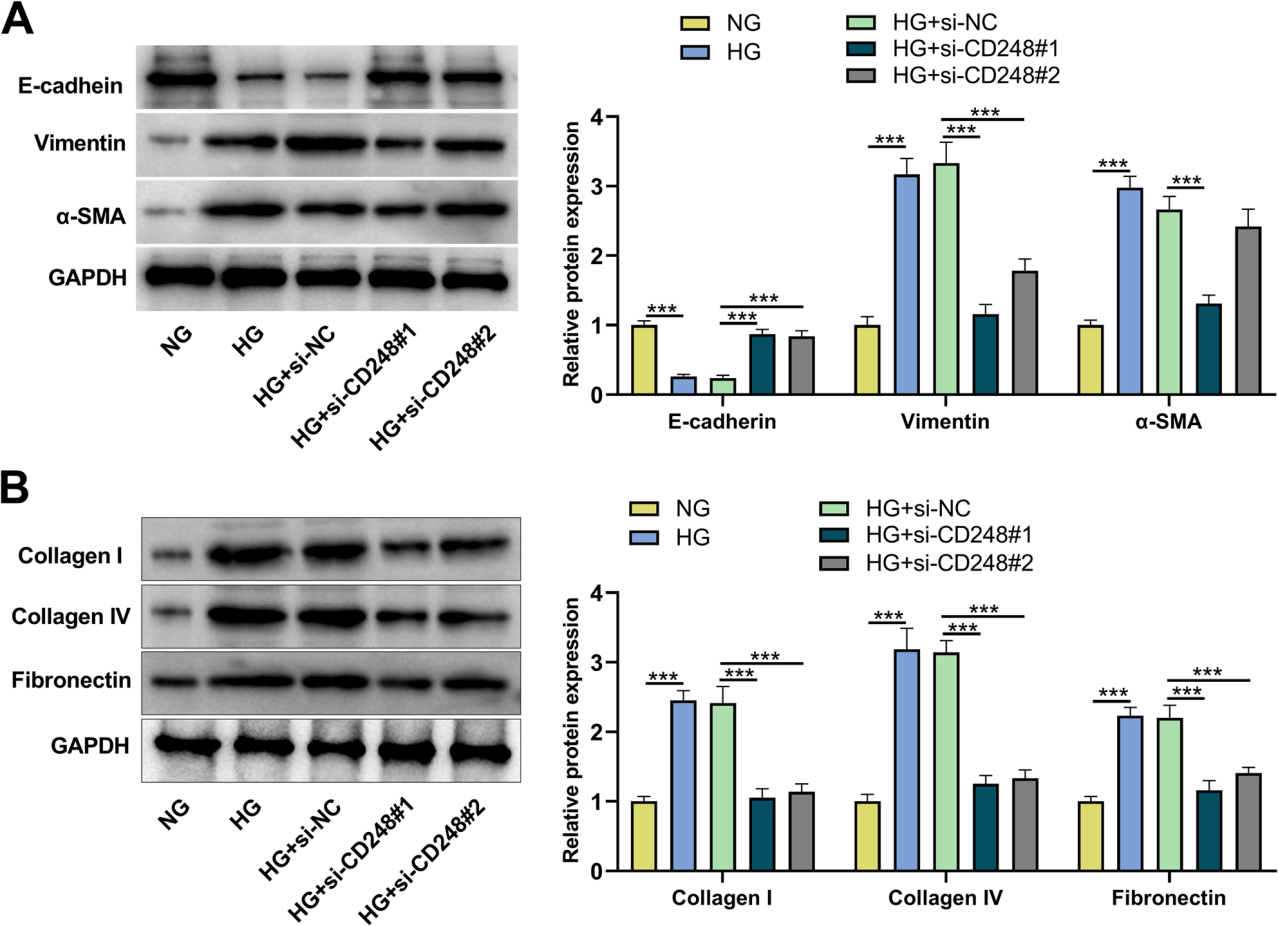
CD248 knockdown inhibited HG-induced EMT and fibrotic changes of HK-2 cells.**A**&**B** HK-2 cells were treated with normal glucose (NG) or high glucose (HG) for 48 h after transfection with si-NC or si-CD248#1/2. Then the protein expression levels of EMT-related markers (E-cadherin, Vimentin and α-SMA) (**A**) and ECM proteins (collagen I, collagen IV and fibronectin) (**B**) were detected by Western blot. Data were presented as mean ± SD (*n* = 3 independent experiments). ***P < 0.001

Furthermore, the effects of CD248 on HG-induced EMT and pro-fibrotic phenotypes of HK-2 cells were investigated. Western blot showed that the expression of epithelial marker E-cadherin was decreased and mesenchymal markers vimentin and α-SMA were increased in the HG group compared with the NG group, while CD248 knockdown hindered HG-induced EMT (Fig. 2A). In addition, HG treatment significantly increased the protein levels of extracellular matrix proteins including collagen I, collagen IV and fibronectin in HK-2 cells; however, CD248 knockdown decreased protein expression levels of collagen I, collagen IV and fibronectin in HG-induced HK-2 cells (Fig. 2B).

### Effect of CD248 knockdown on TGF-β/Smad signaling pathway in HK-2 cells

**Fig. 3:**
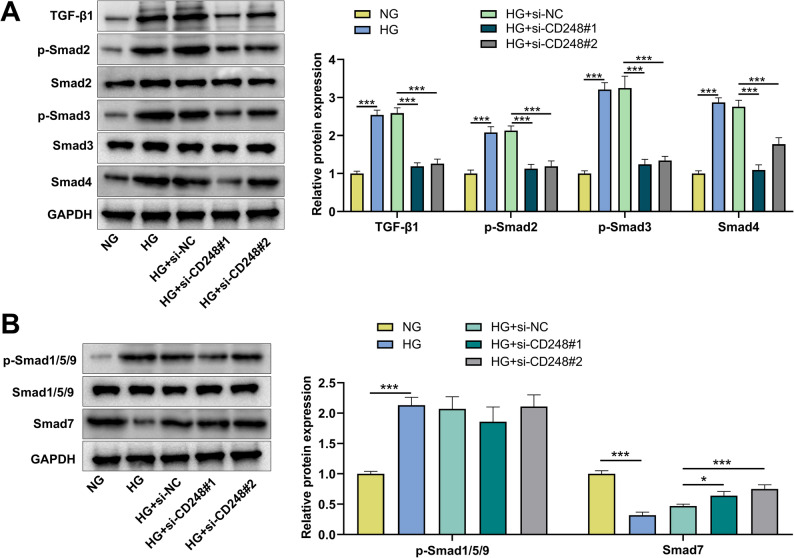
CD248 knockdown inhibits the activation of TGF-β/Smad signaling pathway in HG-induced HK-2 cells.**A**&**B** After transfection with si-NC or si-CD248#1/2, HK-2 cells were treated with normal glucose (NG) or high glucose (HG) for 48 h, and then the protein expression levels of TGF-β1, p-Smad2, p-Smad3, Smad4 (**A**) and p-Smad1/5/9 and Smad7 (**B**) were detected by Western blot. Data were presented as mean ± SD (*n* = 3 independent experiments). *P < 0.05, ***P < 0.001

TGF-β1/Smad signaling pathway plays an important role in renal fibrosis [10]. To further confirm the dependence of CD248’s function on the TGF-β1/Smad pathway, TGF-β1 activator (SRI-011381) rescue experiments were performed. The results showed that the TGF-β1 activator rescued the inhibitory effects of CD248 knockdown on HG-induced TGF-β/Smad signaling activation, cell injury, inflammatory response, EMT and ECM deposition in HK-2 cells, and these findings indicate that the role of CD248 partly depends on the activation of the TGF-β1 pathway (Supplementary Fig. 1A-H). Western blot showed that the protein expression levels of TGF-β1, p-Smad2, p-Smad3 and Smad4 in HG group were significantly increased compared with NG group; at the same time, CD248 knockdown decreased protein expression levels of TGF-β1, p-Smad2, p-Smad3, and Smad4 compared with HG group (Fig. 3A). To rule out the potential cross-talk with the non-canonical BMP signaling pathway, the phosphorylation of Smad1/5/9. While HG stimulation significantly increased the levels of p-Smad1/5/9, CD248 knockdown had no effect on their activation (Fig. 3B). We next assessed the expression of Smad7, a key inhibitory Smad that dampens TGF-β1 signaling [24]. HG treatment markedly downregulated Smad7 protein expression. Intriguingly, knockdown of CD248 effectively restored Smad7 levels (Fig. 3B). These data suggested that CD248 both facilitated the activation of canonical TGF-β/smad pathway, and suppressed the negative feedback.

### Prediction of potential natural drugs targeting CD248

**Fig. 4:**
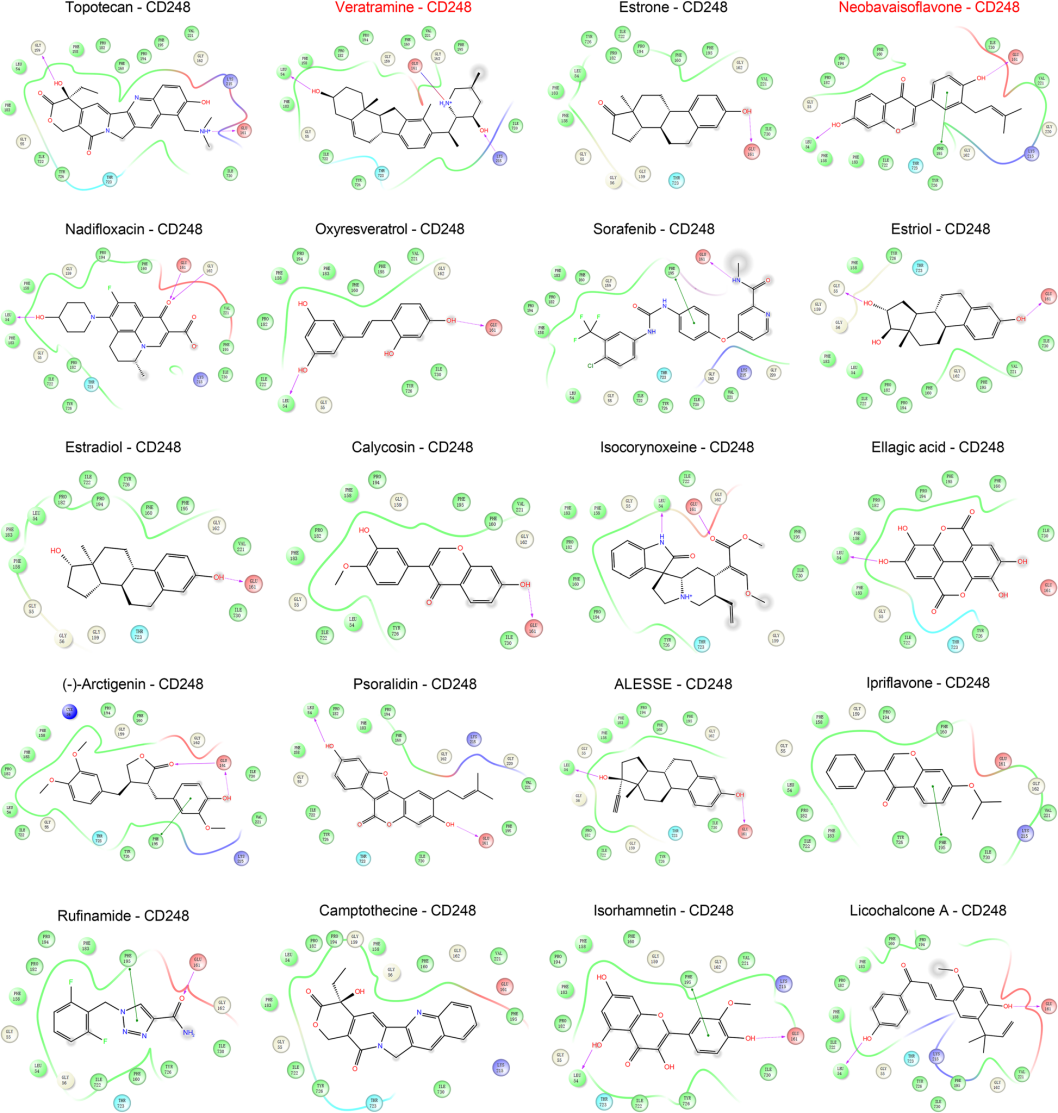
Virtual screening and molecular docking analysis. 2D molecular docking diagram of CD248 protein and topotecan, veratramine (VER), estrone, neobavaisoflavone (NBIF), nadifloxacin, oxyresveratrol, sorafenib, estriol, estradiol, calycosin, isocorynoxeine, ellagic acid, (-)-Arctigenin, psoralidin, ALESSE, ipriflavone, rufinamide, camptothecine, isorhamnetin and Licochalcone A

In order to screen potential natural monomers targeting CD248, virtual screening technology was utilized, and the components were listed according the docking scores with CD248. It was observed that topotecan, VER, estrone, NBIF, nadifloxacin, oxyresveratrol, sorafenib, estriol, estradiol, calycosin, isocorynoxeine, ellagic acid, (-)-Arctigenin, psoralidin could probably stably bind with CD248 protein, and the docking scores were all less than − 7 kcal/mol, indicating that these compounds may be potential drugs targeting CD248 (Table 2; Fig. 4). Considering topotecan is a chemotherapy drug and estrone is a sex hormone, they may lead to potential side effects, thus VER and NBIF were selected for subsequent validation. To further confirm the interaction between VER/NBIF and CD248, the CETSA was performed. The results demonstrated that CD248 exhibited significantly enhanced thermal stability after treatment with VER or NBIF (Supplementary Fig. 2A&B), implying the direct binding between VER/NBIF and CD248.

**Table 2:** Virtual screening and Docking results based on CD248 protein structure

No.	Name	CAS	Docking score	Noncovalent Interactions	Residues
1	Topotecan	123948-87-8	−7.394	2 H–bond	GLY159, GLU161
2	Veratramine	60–70-8	−7.39	1 Salt–bridge, 2 H–bond	GLU161, LYS215, LEU54
3	Estrone	53-16-7	−7.317	1 H–bond	GLU161
4	Neobavaisoflavone	41060-15-5	−7.317	1 π-π stacking, 2 H–bond	PHE195, GLU161, LEU54
5	Nadifloxacin	124858-35-1	−7.224	3 H–bond	GLU161, GLY162, LEU54
6	Oxyresveratrol	29700-22-9	−7.223	2 H–bond	LEU54, GLU161
7	Sorafenib	284461-73-0	−7.218	1 π-π stacking, 1 H–bond	PHE195, GLU161
8	Estriol	50-27-1	−7.211	2 H–bond	GLU161, GLY55
9	estradiol	50-28-2	−7.161	1 H–bond	GLU161
10	Calycosin	20575-57-9	−7.136	1 H–bond	GLU161
11	Isocorynoxeine	51014-29-0	−7.086	2 H–bond	GLU161, LEU54
12	Ellagic acid	476-66-4	−7.057	1 H–bond	LEU54
13	(-)-Arctigenin	7770-78-7	−7.014	1 π-π stacking, 2 H–bond	PHE195, GLU161
14	Psoralidin	18642-23-4	−7.004	2 H–bond	GLU161, LEU54
15	ALESSE	57–63-6	−7	2 H–bond	GLU161, LEU54
16	Ipriflavone	35212-22-7	−6.984	1 π-π stacking	PHE195
17	Rufinamide	106308-44-5	−6.976	1 π-π stacking, 1 H–bond	PHE195, GLU161
18	Camptothecine	2,114,454	−6.965	-	-
19	Isorhamnetin	480-19-3	−6.908	1 π-π stacking, 2 H–bond	PHE195, GLU161, LEU54
20	Licochalcone A	58749-22-7	−6.907	2 H–bond	GLU161, LEU54

### Effect of VER or NBIF therapy on HG-induced HK-2 cell apoptosis and inflammation

**Fig. 5:**
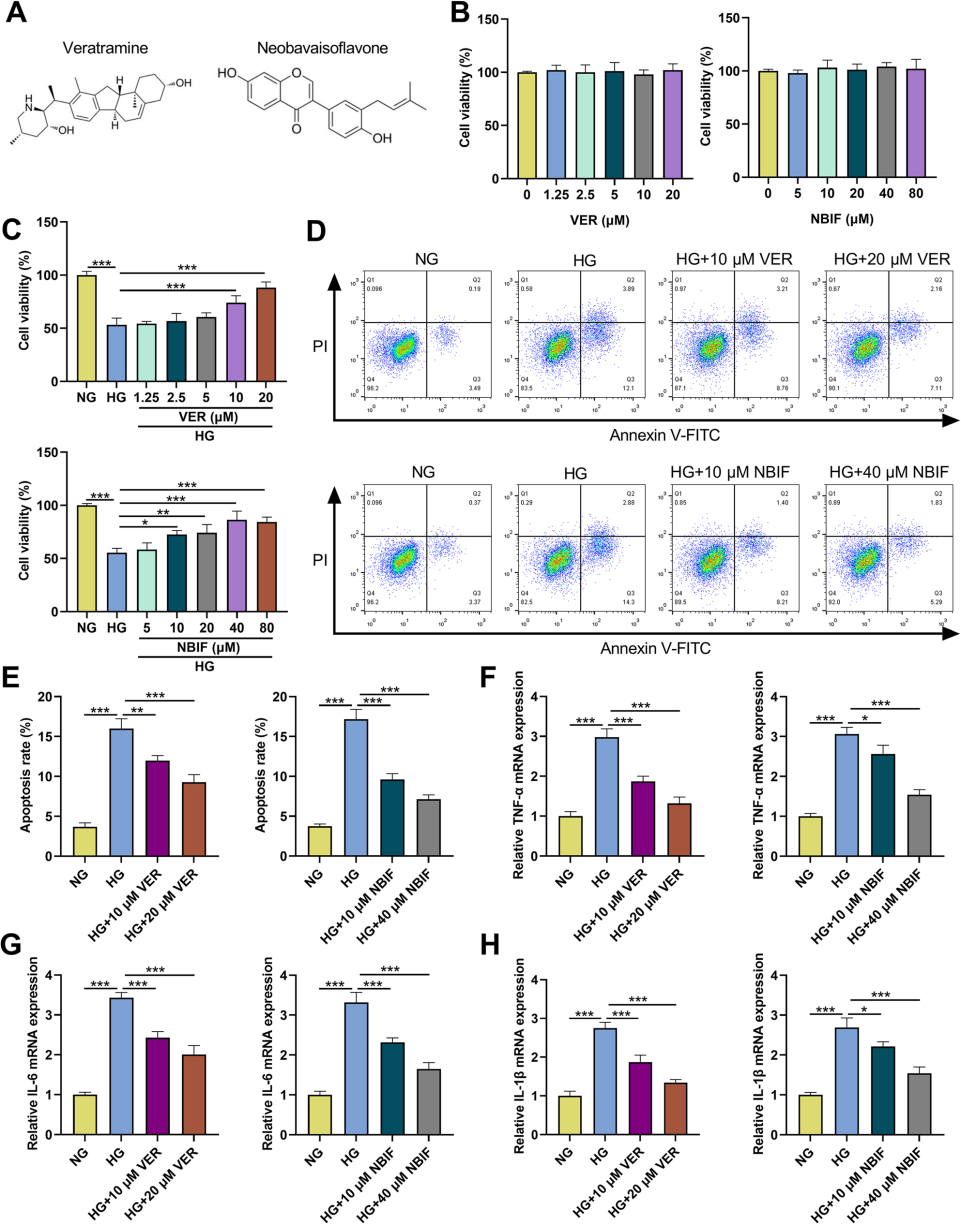
Effects of VER or NBIF treatment on HG-induced apoptosis and inflammation of HK-2 cells.**A** 2D chemical structure of VER and NBIF. **B** HK-2 cells were treated with different concentrations of VER (0, 1.25, 2.5, 5, 10, and 20 µM) or different concentrations of NBIF (0, 5, 10, 20, 40, and 80 µM) for 24 h under normal glucose (NG) conditions, and then the cell viability was detected by CCK-8 assay. **C** HK-2 cells were pretreated with different concentrations of VER (0, 1.25, 2.5, 5, 10, and 20 µM) or different concentrations of NBIF (0, 5, 10, 20, 40, and 80 µM) for 24 h and then treated with NG or high glucose (HG) for 48 h, and then the cell viability was detected by CCK-8 assay. **D**-**H** HK-2 cells were pretreated with 10 µM and 20 µM VER or 10 µM and 40 µM NBIF for 24 h and then treated with NG or HG for 48 h, and flow cytometry was used to assess apoptosis (D&E), and the mRNA expression levels of TNF-α, IL-6 and IL-1β were detected by RT-qPCR (**F**-**H**). Data were presented as mean ± SD (*n* = 3 independent experiments). *P < 0.05, **P < 0.01, and ***P < 0.001

The chemical structures of VER and NBIF are shown in Fig. 5A. To investigate the potential therapeutic effect of VER or NBIF on DN, at first, HK-2 cells were treated with different concentrations of VER (0, 1.25, 2.5, 5, 10, and 20 µM) or different concentrations of NBIF (0, 5, 10, 20, 40, and 80 µM) for 24 h, and then the cell viability was assessed using CCK-8 assay. The results showed that there was no significant effect on cell viability when HK-2 cells were treated with VER or NBIF under NG conditions (Figs. 5B). Furthermore, we examined the effects of VER and NBIF on HG-induced suppression of HK-2 cell viability. The results demonstrated that VER at concentrations ≥ 10 µM significantly reversed the HG-induced inhibition of HK-2 cell viability, while NBIF, within the concentration range of 10–80 µM, markedly enhanced the viability of HG-induced HK-2 cells, with the most pronounced effect observed at 40 µM (Fig. 5C). Based on these findings, subsequent experiments used VER at 10 µM and 20 µM (low and high doses, respectively) and NBIF at 10 µM and 40 µM (low and high doses, respectively) for further investigation. Flow cytometry showed that VER or NBIF treatment dose-dependently repressed HG-induced apoptosis of HK-2 cells (Figs. 5D&E). To investigate whether the effects of VER and NBIF were entirely dependent on CD248, we knocked down CD248 using si-CD248#1 in HK-2 cells and treated them with VER or NBIF under HG condition. Compared to the si-CD248 + DMSO group, treatment with VER or NBIF in the CD248-knockdown background (si-CD248 + VER and si-CD248 + NBIF groups) led to a further enhancement in cell viability (Supplementary Fig. 3 A), and a further decrease in apoptosis (Supplementary Fig. 3B). These results demonstrated that the mechanisms of action of VER and NBIF were not solely dependent on CD248 and were likely to involve additional targets or pathways. In addition, mRNA expression levels of TNF-α, IL-6, and IL-1β were increased in HG-induced HK-2 cells, whereas treatment with VER or NBIF significantly reduced the mRNA expression levels of these pro-inflammatory cytokines in a dose-dependent manner (Figs. 5F-H). These findings indicate that treatment with VER or NBIF alleviates HG-induced apoptosis and inflammatory responses in renal tubular epithelial cells.

### Effect of VER or NBIF therapy on HG-induced HK-2 cell EMT and fibrotic changes

**Fig. 6:**
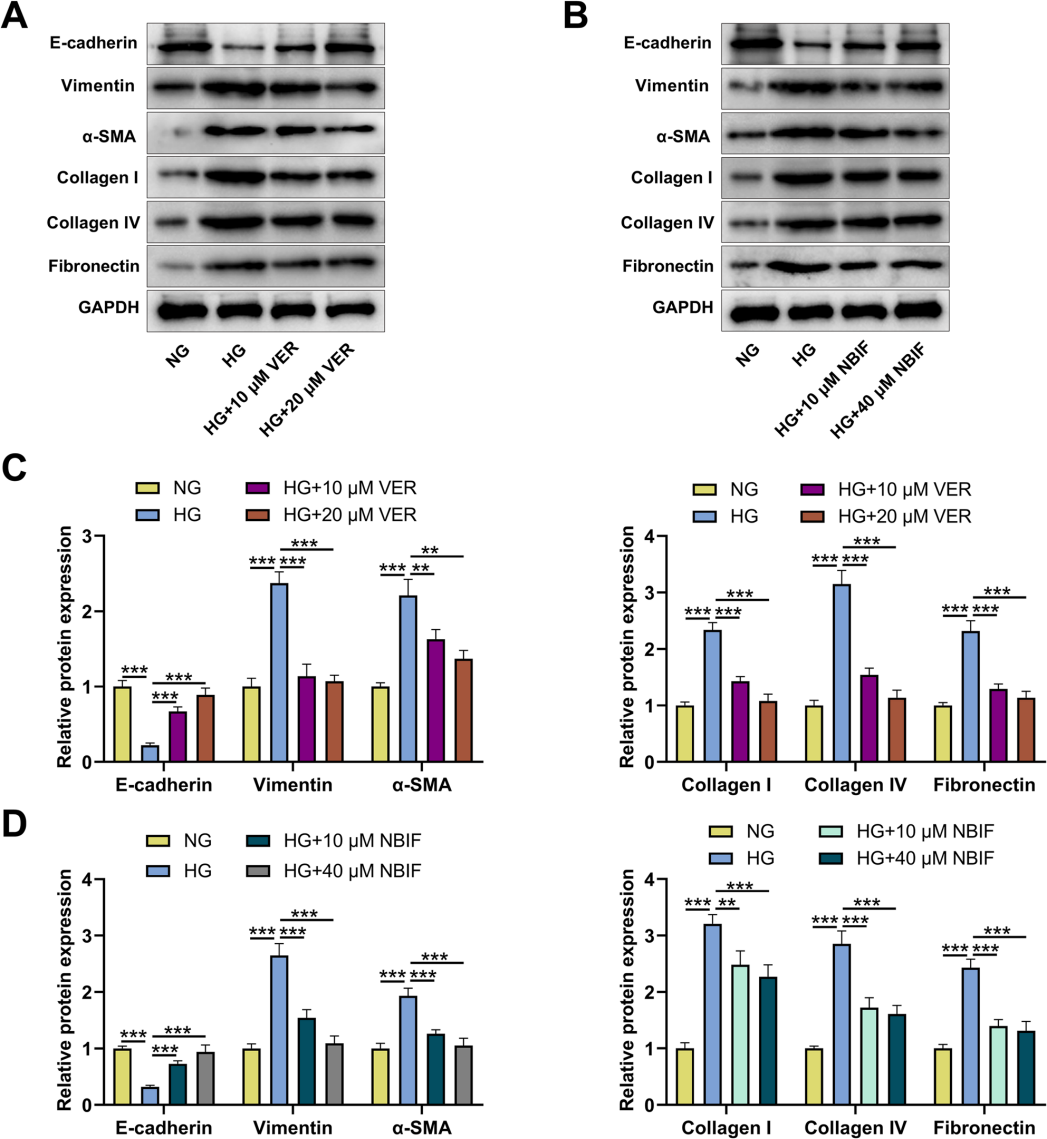
Effects of VER or NBIF treatment on EMT and fibrotic changes in HG-induced HK-2 cells.**A**-**D** HK-2 cells were pretreated with 10 µM and 20 µM VER (A&C) or 10 µM and 40 µM NBIF (B&D) for 24 h and then treated with normal glucose (NG) or high glucose (HG) for 48 h, and the protein expression levels of E-cadherin, Vimentin and α-SMA, and collagen I, collagen IV and fibronectin in HK-2 cells were detected by Western blot. Data were presented as mean ± SD (*n* = 3 independent experiments). **P < 0.05, ***P < 0.001

Next, we investigated the effects of VER or NBIF on HG-induced EMT and fibrosis in HK-2 cells. Western blot showed that compared with the HG group, treatment with VER or NBIF significantly increased the protein level of E-cadherin and decreased the protein levels of Vimentin, α-SMA, collagen I, collagen IV, and fibronectin in HK-2 cells in a dose-dependent manner (Figs. 6A-D). This suggests that VER or NBIF can inhibit HG-induced EMT and fibrosis in renal tubular epithelial cells.

### VER or NBIF inhibited the activity of CD248/TGF-β/Smad axis in HG-induced HK-2 cells

**Fig. 7:**
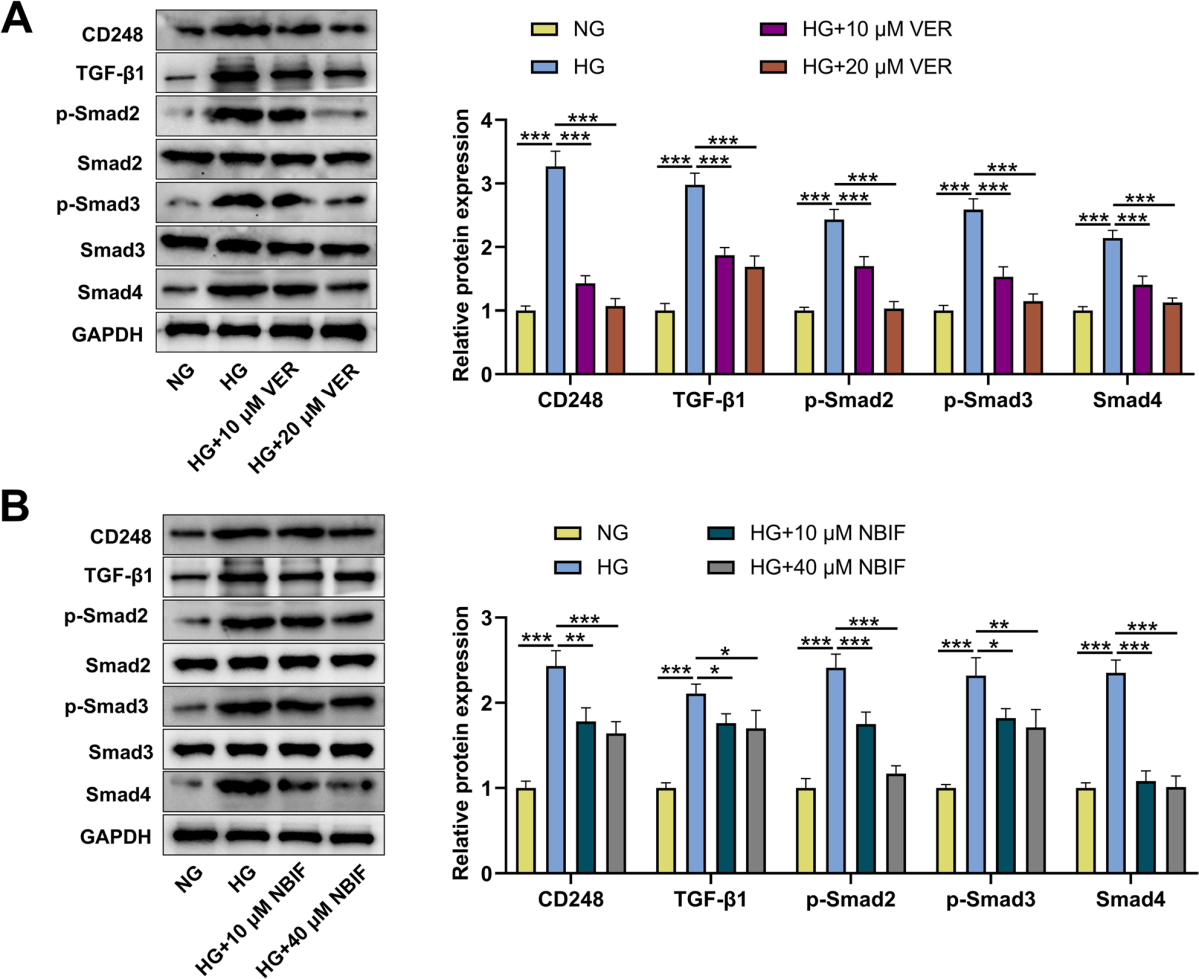
Effect of VER or NBIF treatment on TGF-β/Smad axis in HG-induced HK-2 cells.**A**&**B** HK-2 cells were pretreated with 10 µM and 20 µM VER (**A**) or 10 µM and 40 µM NBIF (**B**) for 24 h and then treated with normal glucose (NG) or high glucose (HG) for 48 h. The protein expression levels of CD248, TGF-β1, p-Smad2, p-Smad3 and Smad4 in HK-2 cells were then detected by Western blot. Data were presented as mean ± SD (*n* = 3 independent experiments). *P < 0.05, **P < 0.01, and ***P < 0.001

To explore the molecular mechanism of VER or NBIF in anti-DN, the effects of VER or NBIF treatment on the CD248/TGF-β/Smad axis in HG-induced HK-2 cells were investigated. Western blot showed that HG treatment increased protein expression levels of CD248, TGF-β1, p-Smad2, p-Smad3, and Smad4 in HK-2 cells, while VER or NBIF treatment reversed these effects in a dose-dependent manner (Figs. 7A and B). This suggests that VER or NBIF may be involved in inhibiting the progression of renal fibrosis in DN by regulating the CD248/TGF-β/Smad axis.

### VER and NBIF ameliorate renal injury, inflammation, and fibrosis in db/db mice

**Fig. 8:**
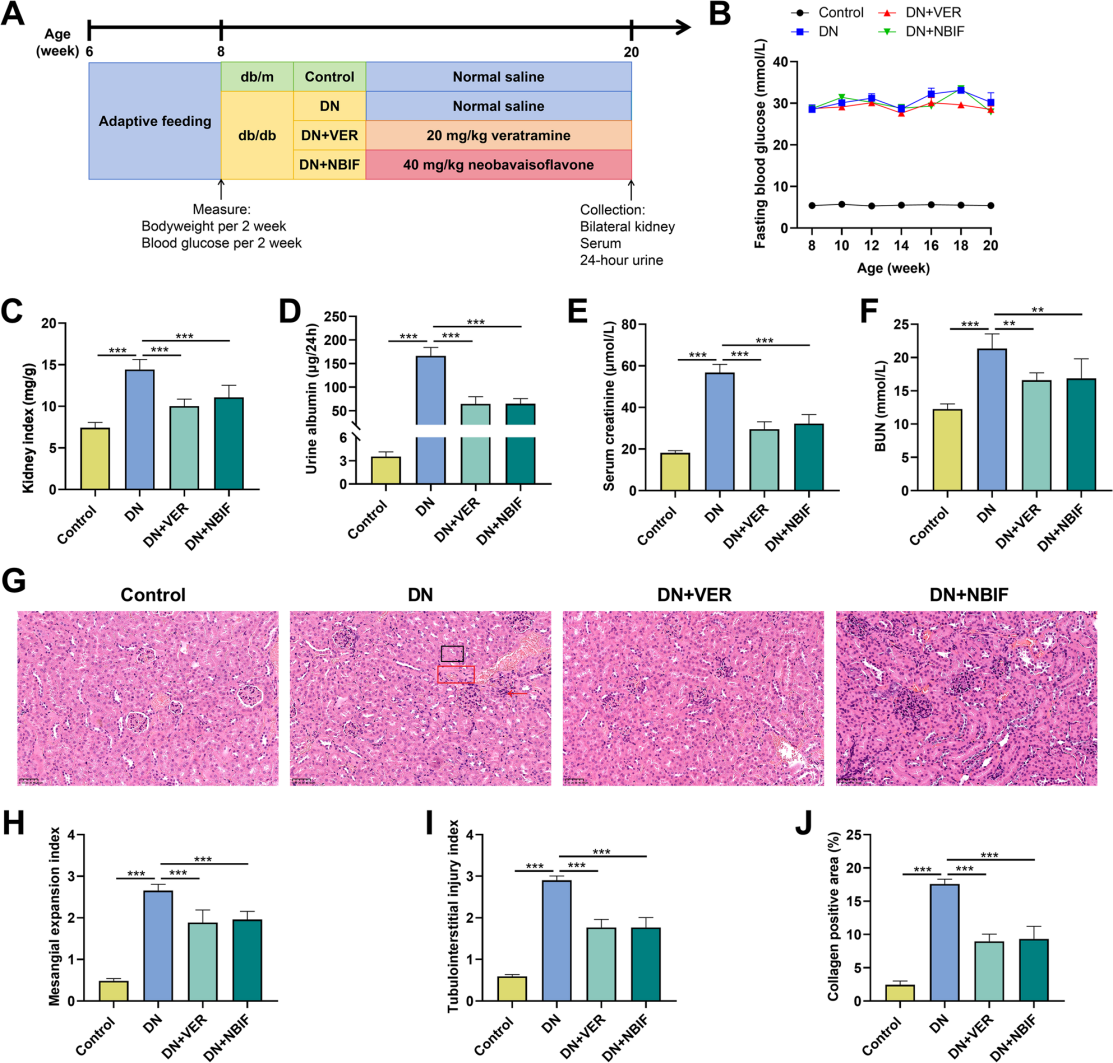
Effects of VER or NBIF on biochemical parameters and renal pathology in db/db mice.**A** The workflow of the animal experiment. **B**-**F** Fasting blood glucose (FBG) levels (**B**), kidney index (**C**), 24 h urine albumin (**D**), serum creatinine (**E**), serum BUN (**F**) of the animals before the euthanasia were measured in different groups to evaluate kidney injury of the animals. **G** Representative images of H&E staining. The black square, vacuolate degeneration of renal tubular epithelial cell; red arrow, inflammatory cell infiltration; the red square, deposition of fibrous tissues. Scale bars = 50 μm. **H**-**J** Mesangial expansion index (**H**), tubulointerstitial injury index (**I**) and collagen deposition were evaluated by pathologists to evaluate kidney injury of the animals in different groups. Data are presented as mean ± SD, *n* = 6. **P < 0.05, ***P < 0.001

**Fig. 9:**
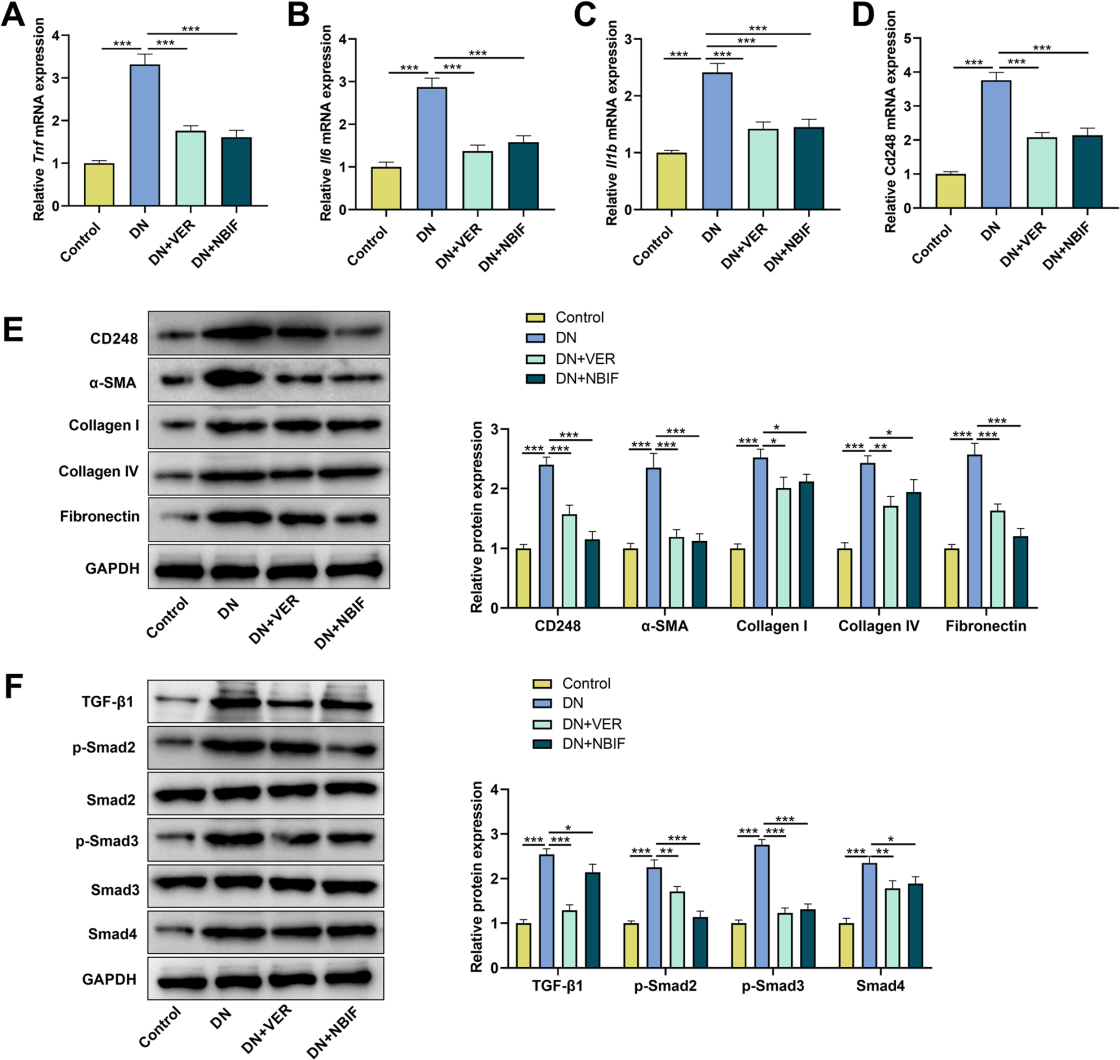
VER and NBIF alleviates inflammation, ECM deposition, and inhibits the activation of the TGF-β1/Smad pathway in db/db mice. **A**-**D** The mRNA expression levels of TNF-α, IL-6, IL-1β, and CD248 in the kidney tissues of the animals in different groups were detected by qPCR. **E**-**H** The protein levels of CD248, ECM proteins (α-SMA, Collagen I/IV, Fibronectin), and TGF-β1/Smad pathway-related molecules in the kidney tissues of the animals in different groups were detected by Western blot. Data are presented as mean ± SD, *n* = 6. *P < 0.05, **P < 0.01, and ***P < 0.001

To investigate the therapeutic potential of VER and NBIF in vivo, db/db mice were administered VER or NBIF daily via oral gavage (Fig. 8A). It was observed that the animals in the DN group exhibited significantly higher levels of FBG, kidney index, 24-h urinary albumin, serum creatinine, and BUN compared to the control group (Figs. 8B–F). Compared with the DN group, VER or NBIF treatment did not induce significant changes in FBG levels (Fig. 8B). However, both VER and NBIF significantly reduced the kidney index, 24-hour urinary albumin, serum creatinine, and BUN levels in db/db mice (Figs. 8C-F). H&E staining revealed that compared to the control group, mice in the DN group exhibited vacuolate degeneration of renal tubular epithelial cell, inflammatory cell infiltration, and deposition of fibrous tissues; these pathological changes were significantly ameliorated after treatment with VER or NBIF (Figs. 8G-J). qPCR results showed that the mRNA expression levels of pro-inflammatory cytokines (TNF-α, IL-6, IL-1β) and CD248 in the renal tissue of DN group mice were elevated, and these effects were reversed by VER or NBIF treatment (Figs. 9A-D). Western blot demonstrated that the expression of CD248, α-SMA, collagen I, collagen IV, fibronectin, TGF-β1, p-Smad2, p-Smad3, and Smad4 in the kidney tissue of DN group mice was significantly higher than in the control group; however, treatment with VER or NBIF significantly reduced the expression of these proteins in db/db mice (Figs. 9E-H). These results indicate that VER or NBIF treatment restores renal function and alleviates renal tissue damage, inflammation, and fibrosis in diabetic mice.

## Discussion

DN is a serious microvascular complication of DM, which is characterized by glomerular basement membrane thickening, mesangial structure changes, podocyte injury and excessive accumulation of ECM proteins, leading to renal fibrosis [25]. So far, there is no specific treatment that can block fibrosis during DN [26]. CD248, as a type I transmembrane glycoprotein, is up-regulated in mouse models of renal fibrosis [19]. Gene deficiency of CD248 has been reported to inhibit microvascular thinning and renal fibrosis [27]. CD248 is also identified as a pivotal stromal cell marker in renal allograft rejection, associated with HIF-1α and IL-1β pathways (also two pivotal pathways in renal fibrosis of DN) [28]. A recent study reports that, in the kidney tissues, elevated CD248 expression is observed in the mesangial area of DN patients, and in the db/db mice, eosinophilic infiltration was noted within the renal interstitium, and the extent of such infiltration exhibited a positive correlation with CD248 expression [29]. However, the role and mechanism of CD248 in renal tubular epithelial cells in DN remain to be clarified.

Glucose level is an independent risk factor for DN development [25]. In HG state, tubular structure and function changes precede glomerular injury, and the increased reabsorption and secretion of glucose by tubular cells leads to tubular hypertrophy, which is one of the earliest manifestations of DN [30]. It has been reported that HG stimulation can promote the expression of some essential fibrosis factors, further increase the excessive accumulation of ECM proteins, and accelerate EMT and renal fibrosis [25, 31]. More than one-third of renal interstitial myoblasts originate from renal tubular epithelial cells [30–32]. Previous studies also report that inhibition of the EMT of renal tubular epithelial cells can significantly ameliorate renal fibrosis in DN [12, 25, 33]. Therefore, intervention targeting renal tubular epithelial cells may become a new strategy for DN treatment. In this study, we found that CD248 was significantly up-regulated in HG-treated human proximal renal tubular epithelial cells (HK-2). In addition, consistent with previous findings [22, 25, 34], HG stimulation can inhibit the proliferation of HK-2 cells, promote apoptosis and inflammatory response, and induce EMT and pro-fibrotic phenotype of HK-2 cells. Importantly, this study demonstrated that CD248 knockdown inhibited HG-induced HK-2 cell damage and inhibited HG-induced EMT and pro-fibrotic phenotype. These findings suggest that CD248 in renal tubular epithelial cells may play an important role in DN development.

TGF-β is considered to be a key regulatory factor driving renal fibrosis. Three subtypes of the TGF-β family have been identified in mammals: TGF-β1, TGF-β2, and TGF-β3. Compared to other subtypes, TGF-β1 is produced in all types of kidney cells [35]. Some evidence suggests that the TGF-β1/Smad signaling pathway is highly upregulated or activated in DN patients and animal models [36, 37]. TGF-β1 can extensively stimulate the phosphorylation of Smad2 and Smad3, and then activate Smad2 and Smad3 to bind to Smad4 to form oligomer complexes, which translocate to the nucleus to regulate the transcription of target genes (such as collagen I, collagen IV and fibronectin) [38]. Therefore, TGF-β1 and Smads are considered therapeutic targets for renal fibrosis. It has been reported that HG stimulation can increase the expression of TGF-β1 in HK-2 cells and activate downstream Smad2, Smad3 and Smad4 [31, 39], which is consistent with the results of this study. Notably, a recent study has reported that CD248 activates TGF-β1 signaling by interacting with and stabilizing transforming growth factor β receptor 2 (TGFBR2) in dermal fibroblast [40]. Interestingly, our study found that CD248 knockdown significantly reduced protein levels of TGF-β1, p-Smad2, p-Smad3, and Smad4 in HG-induced HK-2 cells. This suggests that CD248 may be involved in the development of DN renal fibrosis through TGF-β1/Smad pathway.

Natural products have historically been a valuable source for the discovery and development of various drugs [41]. Therefore, the search for effective antagonists targeting CD248 from natural products is of great significance for the prevention and treatment of DN. VER is a known natural steroid alkaloid found in various plants of the Liliaceae family [42] and has been shown to be effective in lowering blood pressure, antagonizing Na + channel activity, and having agonist activity [43]. Previous studies have shown that VER induces autophagy death by blocking the PI3K/Akt/mTOR signaling pathway, and inhibits the growth of hepatoma HepG2 cells in vitro and in vivo [44]. VER can also relieve pain symptoms in diabetic peripheral neuropathy rats by inhibiting the activation of SIGMAR1-NMDAR pathway [45]. NBIF is a natural flavonoid isolated from the seeds of psoralea and has anti-inflammatory, anti-cancer and antioxidant properties. NBIF showed significant anti-inflammatory effects in activated RAW264.7 macrophages [46]. NBIF has been reported to induce pyroptosis of hepatocellular carcinoma cells [47], and to ameliorate medial collateral ligament-induced osteoarthritis by inhibiting the NF-κB/HIF-2α axis [48]. However, the phamaceutical values of VER and NBIF in DN remain unclear. It is worth mentioning that in this study, VER and NBIF were found to stably bind with CD248 protein, suggesting that they may be potential compounds targeting CD248. In vitro and in vivo assays showed that VER or NBIF therapy could reverse HG-induced inhibition of HK-2 cell proliferation, apoptosis, and inflammation, and could block the progression of HG-induced EMT and fibrosis, and ameliorate renal injury of db/db mice; in addition, VER or NBIF treatment could also inhibited activation of the TGF-β1/Smads axis in vitro and in vivo. These results suggest that VER or NBIF may be potential compounds for the treatment of DN, and its therapeutic effect is related to the CD248/TGF-β1/Smads pathway.

This study has several limitations. First, the findings lack validation using patient-derived specimens. In subsequent studies, the collection of biopsy tissues from DN patients and the analysis of CD248 expression in renal tubular epithelial cells can help further clarify the function of CD248 in the phenotype transformation of renal tubular epithelial cells in HG condition. Secondly, the functional analysis of CD248 relied on siRNA-mediated knockdown, which only transiently and partially suppresses gene expression, unlike the complete and permanent knockout achievable with CRISPR/Cas9; and in the present work, gain-of-function assays of CD248 were also not designed. These techniques/models deserve to be applied in the following study, to further explore the role of CD248 in DN pathogenesis. Thirdly, even though our data support that CD248 modulate TGF-β/Smad pathway, but the detailed molecular mechanism is still obscure, and crucial mediator between CD248 and TGF-β/Smad pathway remains to be identified. Last but not least, the efficacy of natural medicines is often the result of the combined effects of multiple targets and pathways, but it also brings considerable challenges to the quality control. Although this study reports that VER and NBIF can bind to CD248 and inhibit its expression, other downstream targets and signaling pathways of VER and NBIF still need to be further investigated and confirmed. On the other hand, the safe dosage and long-term pharmacological effects of VER and NBIF need to be determined by more pharmacokinetic data based on preclinical studies.

## Conclusion

**Fig. 10:**
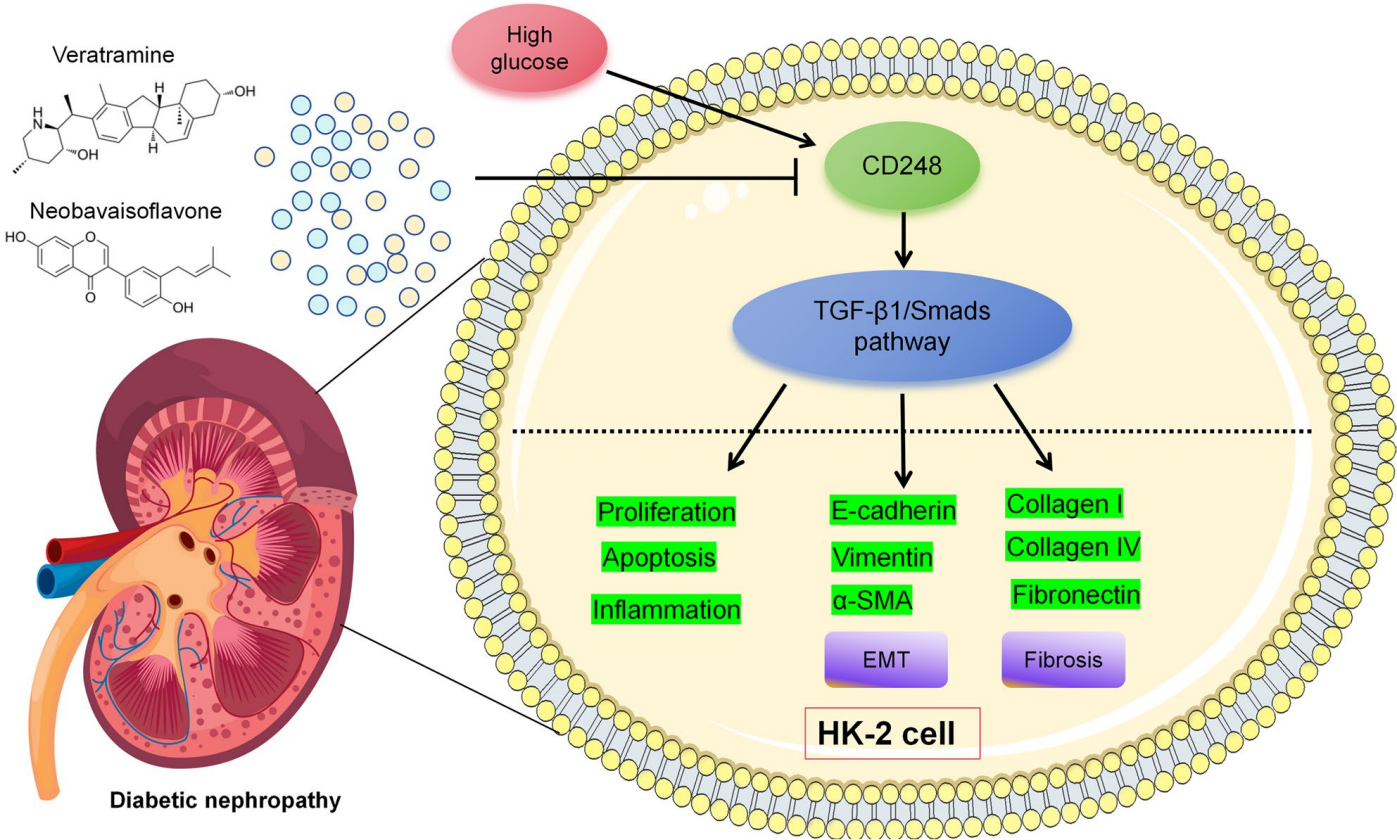
Graphic Abstract: CD248, targeted by veratramine and neobavaisoflavone, mediates pathological changes of renal tubular epithelial cells induced by high glucose

CD248 participates in regulating the proliferation, inflammation, EMT and pro-fibrotic phenotype of renal tubular epithelial cells in HG condition by modulating TGF-β1/Smad pathway. VER and NBIF are potential natural compounds targeting CD248, may be promising drugs for DN treatment (Fig. 10).

## Supplementary Information

Supplementary Material 1. Supplementary figure 1. Activation of TGF-β1 reverses the protective effects of CD248 knockdown on HG-induced injury in HK-2 cells. HK-2 cells were treated under normal glucose (NG) or high glucose (HG) conditions, transfected with si-CD248#1, and/or stimulated with the TGF-β1 activator SRI-011381. A. The protein expression levels of TGF-β1, p-Smad2, p-Smad3, Smad4 were detected by Western blot. B. Cell viability was detected by CCK-8 assay. C. Cell apoptosis was detected by flow cytometry. D. The mRNA expression levels of pro-inflammatory cytokines (TNF-α, IL-6 and IL-1β) were detected by qPCR. E-H. Then the protein expression levels of EMT-related markers (E-cadherin, Vimentin and α-SMA) (E&G) and ECM proteins (collagen I, collagen IV and fibronectin) (F&H) were detected by Western blot. Data were presented as mean ± SD (n = 3 independent experiments). *P<0.05, **P<0.01, and ***P<0.001.

Supplementary Material 2. Supplementary figure 2. VER and NBIF bind directly to CD248 protein in HK-2 cells, as demonstrated by CETSA. A&B. HK-2 cells were treated with DMSO, 20 μM VER (A), or 40 μM NBIF (B) for 4 h. Cell lysates were heated to the indicated temperatures and soluble CD248 protein was detected by Western blot. Data were presented as mean ± SD (n = 3 independent experiments). *P<0.05, **P<0.01, and ***P<0.001. CETSA, cellular thermal shift assay.

Supplementary Material 3. Supplementary figure 3. VER and NBIF ameliorate HG-induced injury in HK-2 cells through both CD248-dependent and independent mechanisms. HK-2 cells were transfected with si-NC or si-CD248 for 48 h, followed by pretreatment with 20 μM VER, 40 μM NBIF, or DMSO for 24 h, and then stimulated with high glucose (HG) for another 48 h. A. Cell viability was detected by CCK-8 assay. B. Cell apoptosis was detected by flow cytometry. Data are presented as mean ± SD (n = 3 independent experiments). **P < 0.05, ***P< 0.001.

Supplementary Material 4.

Figure 1G

Figure 5D

Figure 4

## Data Availability

The data and materials used to support the findings of this study are available from the corresponding author upon request.
