# Supplementary figures and images for: CD248, targeted by veratramine and neobavaisoflavone, mediates pathological changes of renal tubular epithelial cells induced by high glucose
Source: Hereditas. 2025 Dec 12;163:8. doi: 10.1186/s41065-025-00624-z (PMC12781830; doi:10.1186/s41065-025-00624-z)

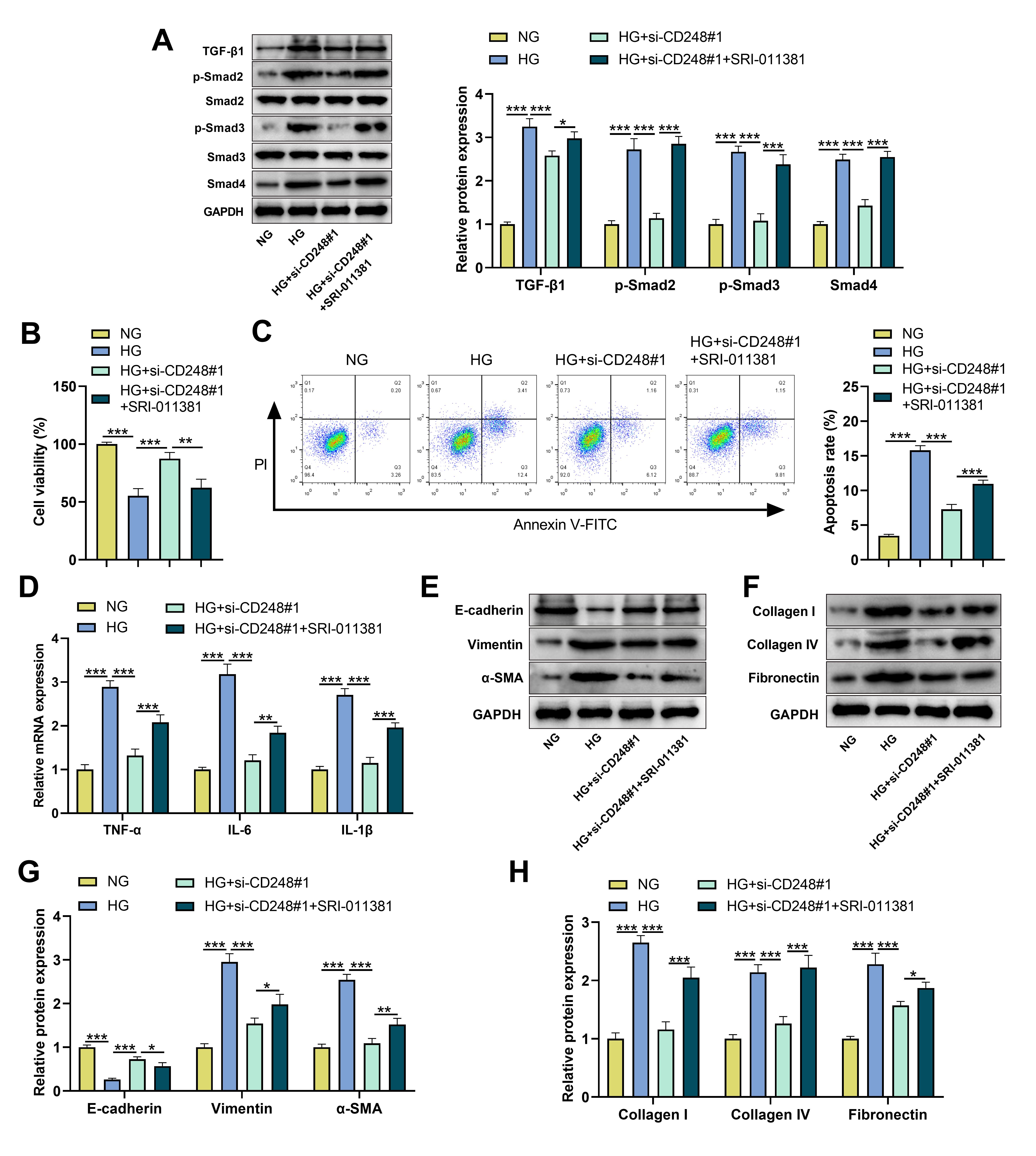

Supplement: Supplementary file 1 — Supplementary Material 1. Supplementary figure 1. Activation of TGF-β1 reverses the protective effects of CD248 knockdown on HG-induced injury in HK-2 cells. HK-2 cells were treated under normal glucose (NG) or high glucose (HG) conditions, transfected with si-CD248#1, and/or stimulated with the TGF-β1 activator SRI-011381. A. The protein expression levels of TGF-β1, p-Smad2, p-Smad3, Smad4 were detected by Western blot. B. Cell viability was detected by CCK-8 assay. C. Cell apoptosis was detected by flow cytometry. D. The mRNA expression levels of pro-inflammatory cytokines (TNF-α, IL-6 and IL-1β) were detected by qPCR. E-H. Then the protein expression levels of EMT-related markers (E-cadherin, Vimentin and α-SMA) (E&G) and ECM proteins (collagen I, collagen IV and fibronectin) (F&H) were detected by Western blot. Data were presented as mean ± SD (n = 3 independent experiments). *P<0.05, **P<0.01, and ***P<0.001. [file 41065_2025_624_MOESM1_ESM.tif]

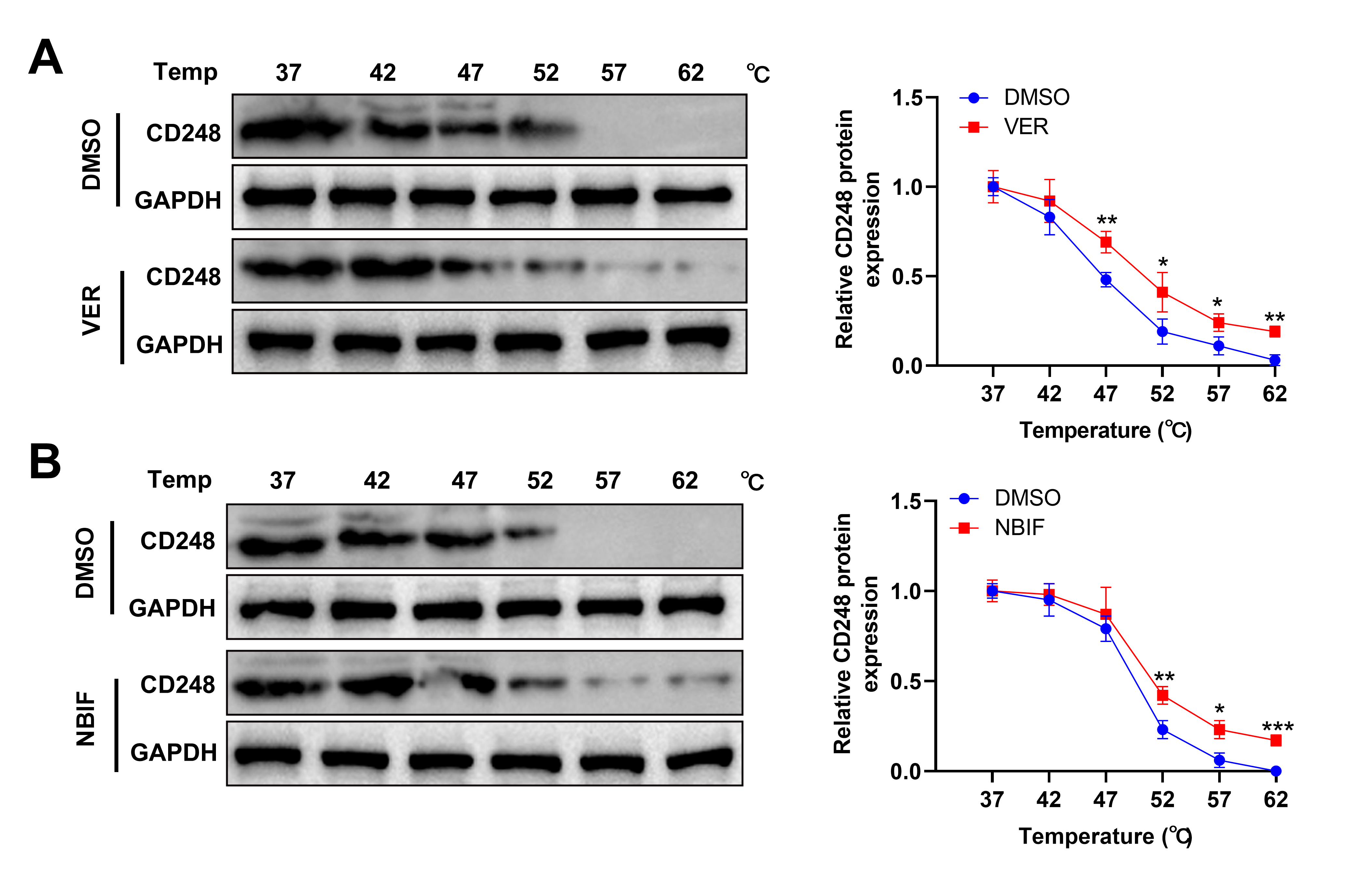

Supplement: Supplementary file 2 — Supplementary Material 2. Supplementary figure 2. VER and NBIF bind directly to CD248 protein in HK-2 cells, as demonstrated by CETSA. A&B. HK-2 cells were treated with DMSO, 20 μM VER (A), or 40 μM NBIF (B) for 4 h. Cell lysates were heated to the indicated temperatures and soluble CD248 protein was detected by Western blot. Data were presented as mean ± SD (n = 3 independent experiments). *P<0.05, **P<0.01, and ***P<0.001. CETSA, cellular thermal shift assay. [file 41065_2025_624_MOESM2_ESM.tif]

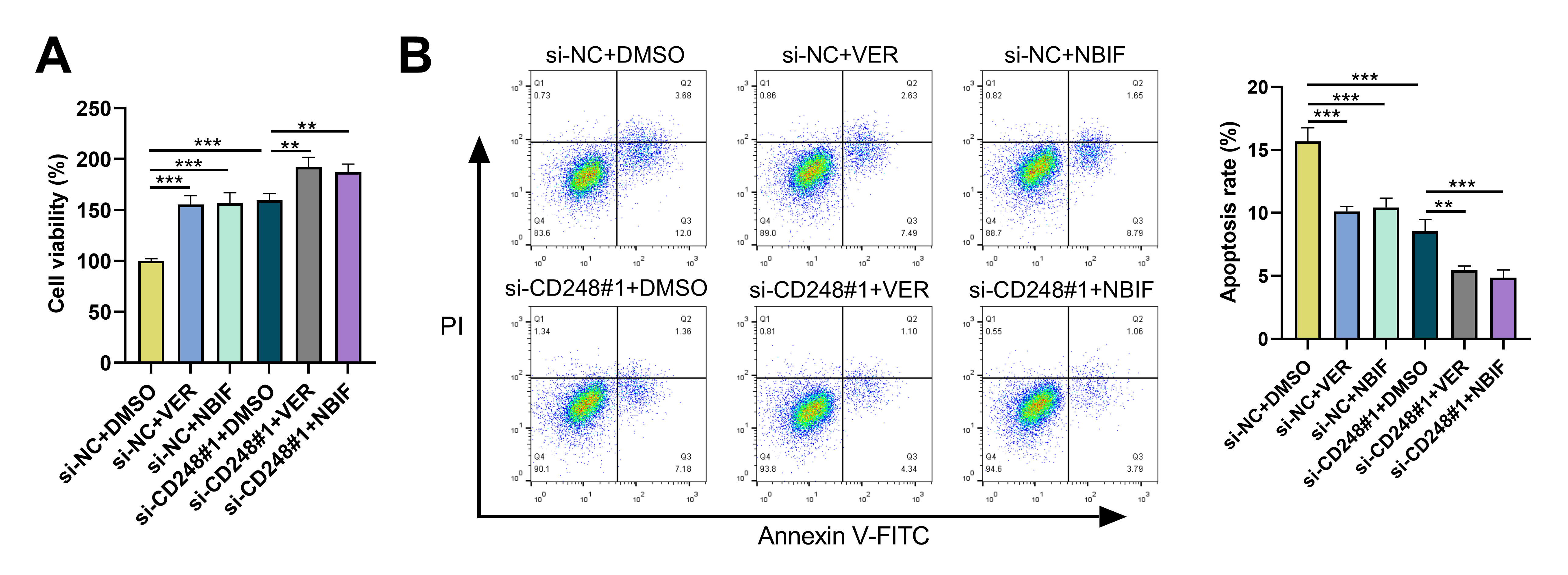

Supplement: Supplementary file 3 — Supplementary Material 3. Supplementary figure 3. VER and NBIF ameliorate HG-induced injury in HK-2 cells through both CD248-dependent and independent mechanisms. HK-2 cells were transfected with si-NC or si-CD248 for 48 h, followed by pretreatment with 20 μM VER, 40 μM NBIF, or DMSO for 24 h, and then stimulated with high glucose (HG) for another 48 h. A. Cell viability was detected by CCK-8 assay. B. Cell apoptosis was detected by flow cytometry. Data are presented as mean ± SD (n = 3 independent experiments). **P < 0.05, ***P< 0.001. [file 41065_2025_624_MOESM3_ESM.tif]
