# Supplementary material for: CD248, targeted by veratramine and neobavaisoflavone, mediates pathological changes of renal tubular epithelial cells induced by high glucose
Source: Hereditas. 2025 Dec 12;163:8. doi: 10.1186/s41065-025-00624-z (PMC12781830; doi:10.1186/s41065-025-00624-z)
Supplement: Supplementary file 4 — Supplementary Material 4. [file 41065_2025_624_MOESM4_ESM.docx]

Original image of Figure 1C CD248

Original image of Figure 1C GAPDH

Original image of Figure 1E CD248

Original image of Figure 1E GAPDH


Original image of Figure 2A E-cadherin

Original image of Figure 2A Vimentin

Original image of Figure 2A α-SMA

Original image of Figure 2A GAPDH


Original image of Figure 2B collagen I

Original image of Figure 2B collagen IV

Original image of Figure 2B fibronectin

Original image of Figure 2B GAPDH


Original image of Figure 3A TGF-β1

Original image of Figure 3A p-Smad2

Original image of Figure 3A Smad2

Original image of Figure 3A p-Smad3

Original image of Figure 3A Smad3

Original image of Figure 3A Smad4

Original image of Figure 3A GAPDH


Original image of Figure 3B p-Smad1/5/9

Original image of Figure 3B Smad1/5/9

Original image of Figure 3B Smad7

Original image of Figure 3B GAPDH


Original image of Figure 6A E-cadherin

Original image of Figure 6A Vimentin

Original image of Figure 6A α-SMA

Original image of Figure 6A collagen I

Original image of Figure 6A collagen IV

Original image of Figure 6A fibronectin

Original image of Figure 6A GAPDH


Original image of Figure 6B E-cadherin

Original image of Figure 6B Vimentin

Original image of Figure 6B α-SMA

Original image of Figure 6B collagen I

Original image of Figure 6B collagen IV

Original image of Figure 6B fibronectin

Original image of Figure 6B GAPDH


Original image of Figure 7A CD248

Original image of Figure 7A TGF-β1

Original image of Figure7A p-Smad2

Original image of Figure 7A Smad2

Original image of Figure 7A p-Smad3

Original image of Figure 7A Smad3

Original image of Figure 7A Smad4

Original image of Figure 7A GAPDH


Original image of Figure 7B CD248

Original image of Figure 7B TGF-β1

Original image of Figure7B p-Smad2

Original image of Figure 7B Smad2

Original image of Figure 7B p-Smad3

Original image of Figure 7B Smad3

Original image of Figure 7B Smad4

Original image of Figure 7B GAPDH


Original image of Supplementary Figure 1A TGF-β1

Original image of Supplementary Figure 1A p-Smad2

Original image of Supplementary Figure 1A Smad2

Original image of Supplementary Figure 1A p-Smad3

Original image of Supplementary Figure 1A Smad3

Original image of Supplementary Figure 1A Smad4

Original image of Supplementary Figure 1A GAPDH


Original image of Supplementary Figure 1E E-cadherin

Original image of Supplementary Figure 1E Vimentin

Original image of Supplementary Figure 1E α-SMA

Original image of Supplementary Figure 1E GAPDH


Original image of Supplementary Figure 1F collagen I

Original image of Supplementary Figure 1F collagen IV

Original image of Supplementary Figure 1Ffibronectin

Original image of Supplementary Figure 1F GAPDH


Original image of Supplementary Figure 2A CD248_DMSO

Original image of Supplementary Figure 2A GAPDH_DMSO

Original image of Supplementary Figure 2A CD248_VER

Original image of Supplementary Figure 2A GAPDH_VER


Original image of Supplementary Figure 2B CD248_DMSO

Original image of Supplementary Figure 2B GAPDH_DMSO

Original image of Supplementary Figure 2B CD248_NBIF

Original image of Supplementary Figure 2B GAPDH_NBIF


Original image of Supplementary Figure5E CD248

Original image of Supplementary Figure5E α-SMA

Original image of Supplementary Figure5E collagen I

Original image of Supplementary Figure5E collagen IV

Original image of Supplementary Figure5E fibronectin

Original image of Supplementary Figure5E GAPDH


Original image of Supplementary Figure5F TGF-β1

Original image of Supplementary Figure5F p-Smad2

Original image of Supplementary Figure5F Smad2

Original image of Supplementary Figure5F p-Smad3

Original image of Supplementary Figure5F Smad3

Original image of Supplementary Figure5F Smad4

Original image of Supplementary Figure5F GAPDH
